# Risk stratification scores for hospitalization duration and disease progression in moderate and severe patients with COVID-19

**DOI:** 10.1186/s12890-021-01487-6

**Published:** 2021-04-14

**Authors:** Jiaqi Huang, Yu Xu, Bin Wang, Ying Xiang, Na Wu, Wenjing Zhang, Tingting Xia, Zhiquan Yuan, Chengying Li, Xiaoyue Jia, Yifan Shan, Menglei Chen, Qi Li, Li Bai, Yafei Li

**Affiliations:** 1 Department of Epidemiology, College of Preventive Medicine, Army Medical University (Third Military Medical University), No. 30 Gaotanyan Street, Chongqing, 400038 People’s Republic of China; 2 Department of Respiratory and Critical Care Medicine, The Second Affiliated Hospital of The Army Medical University, Chongqing, 400037 People’s Republic of China

**Keywords:** COVID-19, Risk stratification score, Disease progression, Length of hospital stay

## Abstract

**Background:**

During outbreak of Coronavirus Disease 2019 (COVID-19), healthcare providers are facing critical clinical decisions based on the prognosis of patients. Decision support tools of risk stratification are needed to predict outcomes in patients with different clinical types of COVID-19.

**Methods:**

This retrospective cohort study recruited 2425 patients with moderate or severe COVID-19. A logistic regression model was used to select and estimate the factors independently associated with outcomes. Simplified risk stratification score systems were constructed to predict outcomes in moderate and severe patients with COVID-19, and their performances were evaluated by discrimination and calibration.

**Results:**

We constructed two risk stratification score systems, named as STPCAL (including significant factors in the prediction model: number of clinical symptoms, the maximum body temperature during hospitalization, platelet count, C-reactive protein, albumin and lactate dehydrogenase) and TRPNCLP (including maximum body temperature during hospitalization, history of respiratory diseases, platelet count, neutrophil-to-lymphocyte ratio, creatinine, lactate dehydrogenase, and prothrombin time), to predict hospitalization duration for moderate patients and disease progression for severe patients, respectively. According to STPCAL score, moderate patients were classified into three risk categories for a longer hospital duration: low (Score 0–1, median = 8 days, with less than 20.0% probabilities), intermediate (Score 2–6, median = 13 days, with 30.0–78.9% probabilities), high (Score 7–9, median = 19 days, with more than 86.5% probabilities). Severe patients were stratified into three risk categories for disease progression: low risk (Score 0–5, with less than 12.7% probabilities), intermediate risk (Score 6–11, with 18.6–69.1% probabilities), and high risk (Score 12–16, with more than 77.9% probabilities) by TRPNCLP score. The two risk scores performed well with good discrimination and calibration.

**Conclusions:**

Two easy-to-use risk stratification score systems were built to predict the outcomes in COVID-19 patients with different clinical types. Identifying high risk patients with longer stay or poor prognosis could assist healthcare providers in triaging patients when allocating limited healthcare during COVID-19 outbreak.

**Supplementary Information:**

The online version contains supplementary material available at 10.1186/s12890-021-01487-6.

## Background

Coronavirus disease 2019 (COVID-19), a newly emerged respiratory disease caused by severe acute respiratory syndrome coronavirus 2 (SARS-CoV-2), has recently become the most important global public health emergency. Because of the coronavirus's novel nature, it remains difficult to come up with specific remedies that will allow us to prevail over COVID-19. It is now widely recognized that a large-scale epidemic of COVID-19 can cause many deaths and more emergency patients, which presents a severe challenge to regional healthcare systems [1]. Rational medical resource allocation and efficiency of emergency rescue, which will be key measures to reduce the mortality of disease, depend on early prediction for length of hospital stay and disease progression.

Among the COVID-19 cases, about 81% are in mild or moderate condition, and 19% are severe or critical cases [2]. Mild patients do not need hospitalization, while some moderate patients may need. The length of hospital stay means the amount of time patients spend on medical resources. Thus, identifying factors affecting hospitalization duration to assess the risk stratification of patients will help to shorten hospital stay to the briefest amount of time possible and alleviate the burden of medical resources. Compared with moderate patients, severe and critical patients are more likely to progress rapidly and have adverse outcomes [3]. Predicting patients at high risk of progression, who often require more care and precise treatment, will improve the prognosis.

A recent systematic review critically appraised published and preprint reports of prediction models for prognosis of patients with COVID-19 [4]. The most reported predictors of severe prognosis included age, sex, C-reactive protein (CRP), lactate dehydrogenase (LDH) and lymphocyte count. However, all included studies were rated at high risk of bias, mostly because of small sample sizes (ranging from 26 to 577 patients) and high risk of model overfitting. Reporting quality varied substantially between studies, and calibration of predictions was rarely assessed. In addition, findings from previous studies are inconsistent. For example, although several studies reported older patients had longer length of hospital stay, other studies have shown that demographic variables including age may not be good indicators for length of stay [5–7]. Therefore, sharing data with large sample sizes and updating of COVID-19 prognosis related prediction models are urgently needed.

Here, we performed a retrospective cohort study in 2425 cases from one of the largest special hospital of COVID-19 in Wuhan, China. Our aims were to construct two risk stratification scoring systems for predicting length of hospital stay and disease progression in moderate and severe patients, respectively. We present the following article in accordance with the STROBE reporting checklist.

## Methods

### Patients

**Fig. 1.**
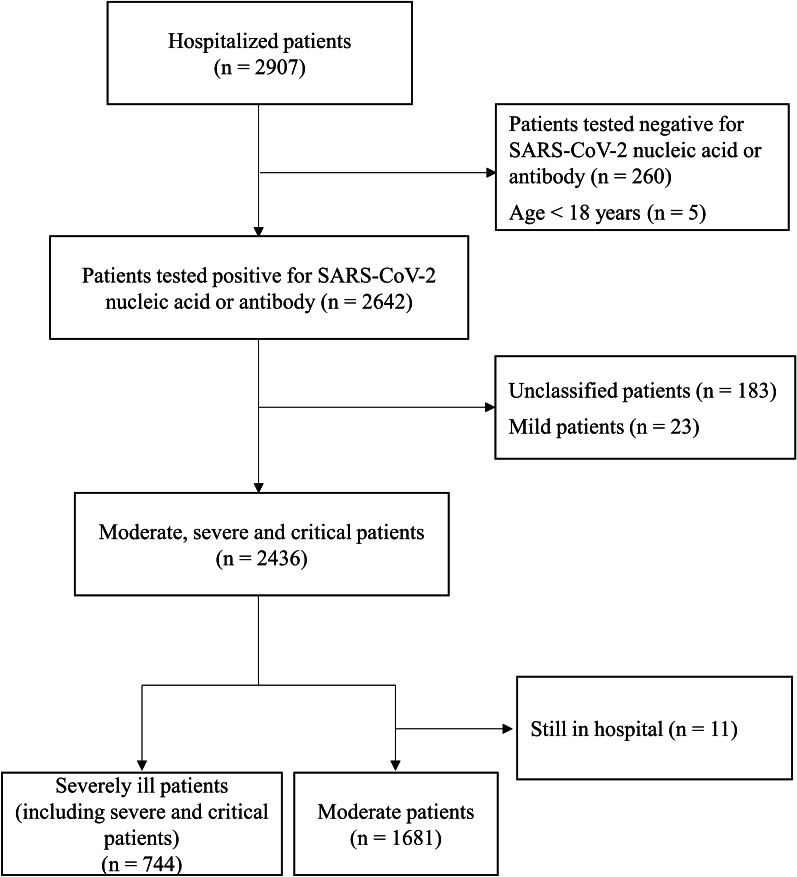
Flow chart for the participants

This retrospective, single-center cohort study was conducted at Huoshenshan Hospital, one of the largest special hospital of COVID-19, in Wuhan, China from January to April 2020. The patient inclusion criteria were at least 18 years old and confirmed with SARS-CoV-2 infection based on positive nucleic acid or antibody detection. Patients with unclassified diagnoses, and moderate patients who had not been discharged by the end of the study were excluded. As of April 14th, 2020, 2907 COVID-19 patients were screened, 265 patients did not meet the inclusion criteria, including 260 cases of negative nucleic acid or antibody test and 5 cases of children or adolescents. Meanwhile, a total of 217 cases were excluded: unclassified or mild type cases (n = 206), moderate patients still in hospital (n = 11). Finally, 2425 of 2642 patients (1681 moderate patients and 744 severely ill patients) were included (Fig. 1). This study was approved by the Ethics Committee of Huoshenshan Hospital.

According to “Diagnosis and Treatment Protocol for Novel Coronavirus Infection-Induced Pneumonia (Version seven)” published by the National Health Commission of China [8]. Mild cases were defined as having mild clinical symptoms (low fever, slight fatigue) and no evidence of pneumonia on imaging, most cases recovered after one week. Mild patients were not included in this study due to the mild symptoms, and majority of them do not need hospitalization. Moderate cases were defined as having symptoms such as fever and respiratory tract symptoms (cough, sore throat, runny nose, and sneezing), etc., with pneumonia. Some cases may have no clinical signs and symptoms, but imaging shows lung lesions. Adult severe cases were defined as meeting any of the following three criteria: (1) respiratory distress, respiratory rate (RR) ≥ 30 times/min; (2) oxygen saturation ≤ 93% at resting state; (3) arterial partial pressure of oxygen (PaO2) / oxygen concentration (FiO2) ≤ 300 mmHg. Critical cases were defined as meeting any of the following criteria: (1) respiratory failure and requiring mechanical ventilation; (2) shock; (3) with other organ failure require Intensive Care Unit (ICU) care. In this study, we combined severe and critical cases as severely ill patients.

The discharge criteria were defined as the following conditions: (1) body temperature returned to normal for at least three days; (2) respiratory symptoms improved obviously; (3) pulmonary imaging showed obvious absorption of inflammation; (4) nucleic acid test was negative for two consecutive times on respiratory tract samples, and the sampling interval was at least 24 hours.

MuLBSTA score, a previous scoring system for predicting the poor prognosis of viral pneumonia, was calculated using following factors: (1) Imaging multiple pulmonary infiltrations (5 points), (2) Lymphocyte counts ≤ 0.8 x10^9^/L (4 points), (3) Combined with bacterial infection (4 points), (4)acute-smoker (3 points)/quit-smoker (2 points), (5) History of hypertension (2 points), (6) Age ≥ 60 years old (2 points). The cut-off value for mortality risk stratification was 12 points [9].

### Data collection

Demographic information, clinical characteristics, radiological data and treatment information of each patient were extracted through the electronic medical record system using a standardized uniform form. Most of treatment measurements were to reduce clinical symptoms and to provide supportive care, such as antibiotics, antiviral, corticosteroids, traditional Chinese medicine, oxygen therapy, etc. More than 85% of patients with SARS-CoV-2 infection are being treated with traditional Chinese medicine in China, such as *Lian Hua Qing Wen Capsule*, *QingfeiPaidu* decoction, *Tan Re Qing* injection,*Xue Bi Jing* injection, etc. These drugs have been recommended as general prescriptions in the diagnosis and treatment protocol of COVID-19 [8, 10].

We also recorded the results of laboratory tests on the peripheral blood of patients within 48 hours after admission. The laboratory biomarkers included blood routine indices [leucocyte count, lymphocyte count, hemoglobin, platelet count, neutrophil-to-lymphocyte ratio (NLR)], infection/inflammation-related indices (CRP), blood biochemistry indices [alanine aminotransferse (ALT), albumin, blood urea nitrogen (BUN), creatinine, creatine kinase, LDH], blood coagulation indices (prothrombin time, D-dimer). All data were checked by two researchers (Yu Xu and Bin Wang) and any disagreement was reached by consensus or participation of third researcher (Li Bai).

### Outcomes

For moderate patients, the length of hospital stay (discharge date minus admission date) was the primary outcome. We used the median of length as the cut-off point to divide moderate patients into short-stay and long-stay groups. For severely ill patients (including severe and critical type), the primary outcome was disease progression, meeting any of the following criteria: from severe to critical or death, from critical to death, or admission to ICU.

### Statistical analysis

Continuous variables were presented by medians with interquartile ranges (IQR), and categorical variables by numbers with percentages. Difference comparisons between groups were performed by a Mann-Whitney U test, Kruskal-Wallis H test or Chi-Square test.

**Fig. 2.**
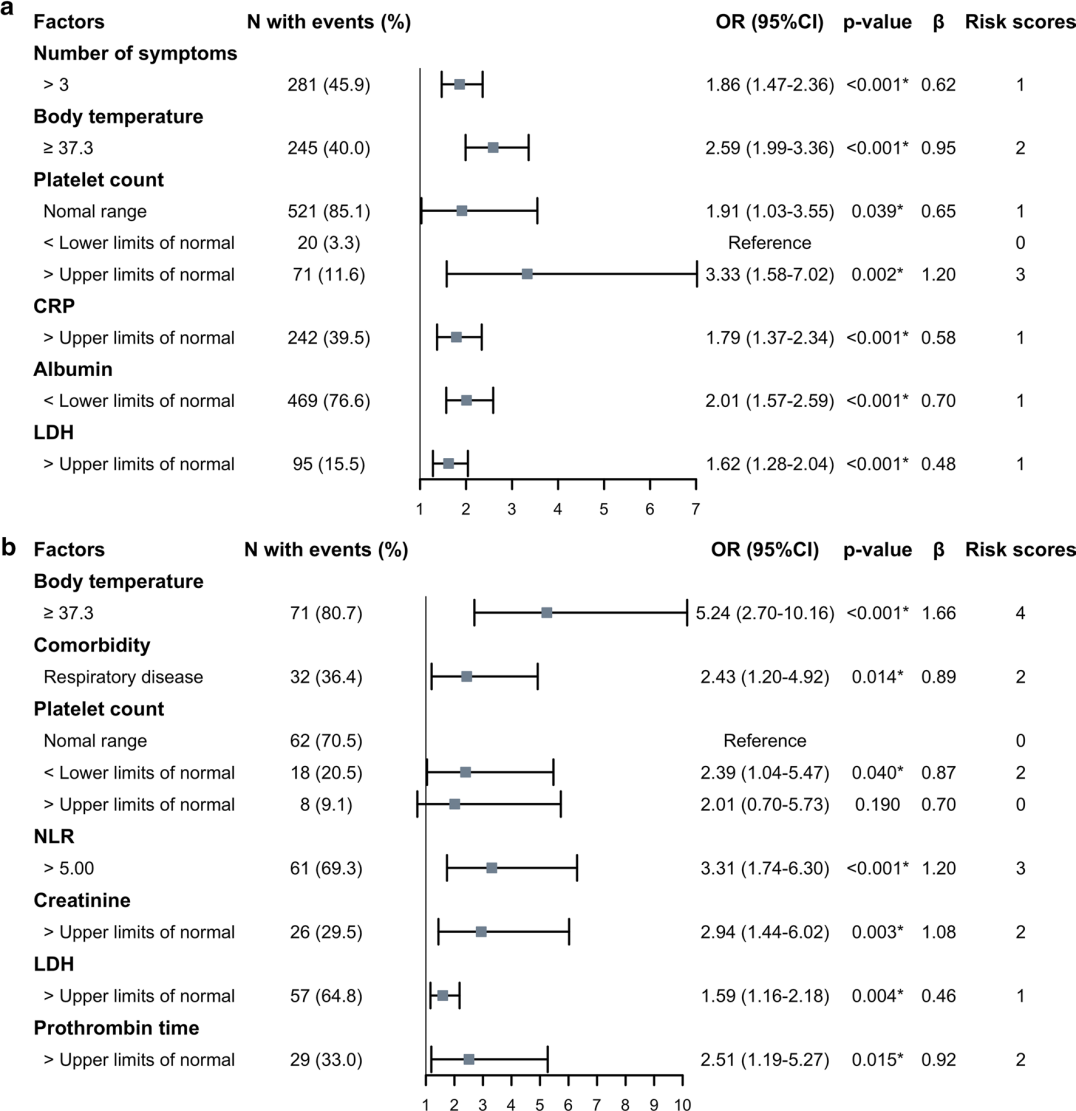
Factors associated with hospitalization duration in moderate patients and disease progression in severely ill patients. **a** Moderated patients. **b** Severely ill patients. CRP, C-reactive protein; LDH, lactate dehydrogenase; NLR, neutrophil-to-lymphocyte ratio. ORs (95% CI) and p-values were calculated using a multivariate logistic regression analysis adjusted the variables with *p* < 0.05 in the univariate logistic regression analyses. * indicates *p* < 0.05. β is the regression coefficient of the multivariate logistic regression model. Risk scores were assigned by the weighted point of factors which simplified by the integer form of the quotient of one factor's regression coefficient and the lowest regression coefficient

A logistic regression analysis was performed to evaluate the independent factors associated with outcomes. In an univariate logistic regression, all laboratory biomarkers were brought in the form of continuous variables. Specific symptoms were replaced by the number of symptoms in this analysis. In a multivariate logistic regression, laboratory biomarkers were defined as categorical variables using the upper or lower limit of normal values (see Additional file 1: Table S1 for details). The cut-off point of NLR was defined by a receiver operator characteristic (ROC) curve (with largest Youden index). A multivariate logistic regression was performed with significant variables (p < 0.05) in the univariate logistic regression. Firstly, the variance inflation factor (VIF) was used to identify collinearity among the covariates. The collinearity was negligible cause the VIFs of variables were less than 2.5. Then three methods (entering, forward and backward for likelihood ratio test) were used to select the significant variables in the multivariate logistic regression. Variables retained in any one of the three method models (with p < 0.05) were used to construct the final model by an entering method (likelihood ratio test). In order to rule out the impact of death on the length of stay of moderate patients, sensitivity analysis was performed to exclude the dead patients. We estimated the goodness of fit of the final model using a Hosmer and Lemeshow test. Risk stratification scores were assigned by the weight of different levels of significant factors. The weighted point (λ) of each factor was simplified by the integer form of the quotient of one factor's regression coefficient and the lowest regression coefficient in the model as shown in Fig. 2 (e.g., number of symptoms > 3 got one point because the quotient of its regression coefficient and LDH's regression coefficient equal to 1.29) [11], and total points were calculated by summing these weighted points.

An internal validation was performed to estimate the predictive performance of risk scores by bootstrapping with 1000 replications of the derivation cohort. The discriminative ability was assessed using the area under the ROC curve (AUC). Discrimination between TRPNCLP and MuLBSTA score was also assessed by comparing AUC, net reclassification improvement (NRI) and integrated discrimination improvement (IDI) for severely ill patients. The calibration for agreement was measured by a calibration-in-the-large (perfect = 0), calibration slope (perfect = 1), and calibration plot after deviation correction [12]. Statistical analysis was performed with SPSS (version 25.0; SPSS Inc., Chicago, IL, USA.) and R (version 3.5.4, R Foundation for Statistical Computing, Vienna, Austria), A two-tailed p-value < 0.05 was considered statistically significant.

## Results

### Risk scoring system to stratify the moderate patients with different length of hospital stay

The demographic and clinical characteristics of moderate patients were summarized in Table 1. The cut-off value of length of hospital stay was defined as 13 days. There were 789 long-stay (> 13 days) patients (50.1% males, median age 61 years) and 892 short-stay patients (49.4% males, median age 56 years). During the observation period, 2 patients with a short-stay and 4 patients with a long-stay died (*p* = 0.332). The main symptoms including fever, cough, fatigue, asthma or dyspnea, and myalgia were more common in long-stay patients than in short-stay (*p* < 0.001). Traditional Chinese medicine (91.2%) and oxygen therapy (60.8%) were widely used; in addition, long-stay patients tended to receive more therapy than short-stay patients did (*p* < 0.001). Compared with short-stay patients, long-stay patients were significantly older, more likely to have higher levels of platelet count, NLR, CRP, ALT, LDH and D-dimer, as well as lower levels of lymphocyte count, hemoglobin, albumin and creatine kinase (*p* < 0.001).

**Table 1.** Demographic and clinical characteristics of moderate patients with COVID-19

	Total (n = 1681)	Length of hospital stays		*p*-value
		Short-stay (≤ 13 days) (n = 892)	Long-stay (> 13 days) (n = 789)	
**Age (years)**	58 (48,67)	56 (46, 65)	61 (50, 68)	**<0.001**
≥ 60	786 (46.8%)	370 (41.5%)	416 (52.7%)	**<0.001**
**Gender**				
Male	836 (49.7%)	441 (49.4%)	395 (50.1%)	0.799
Female	845 (50.3%)	451 (50.6%)	394 (49.9%)	
**History of smoking**				
Yes	115/1662 (6.9%)	66/885 (7.5%)	49/777 (6.3%)	0.356
**Clinical symptoms**				
Cough	1182 (70.3%)	584 (65.5%)	598 (75.8%)	**<0.001**
Fever	1216 (72.3%)	585 (65.6%)	631 (80.0%)	**<0.001**
Asthma or dyspnea	702 (41.8%)	311 (34.9%)	391 (49.6%)	**<0.001**
Fatigue	898 (53.4%)	430 (48.2%)	468 (59.3%)	**<0.001**
Myalgia	500 (29.7%)	217 (24.3%)	283 (35.9%)	**<0.001**
Other features	464 (27.6%)	235 (26.3%)	229 (29.0%)	0.220
**Clinical outcome**				
Discharge	1675 (99.6%)	890 (99.8%)	785 (99.5%)	0.332
Died	6 (0.4%)	2 (0.2%)	4 (0.5%)	
**Number of symptoms**	3 (2, 4)	3 (2, 4)	3 (2, 5)	**<0.001**
> 3	644 (38.3%)	272 (30.5%)	372 (47.1%)	**<0.001**
**Maximum body temperature ≥ 37.3 ℃**	502/1664 (30.2%)	167/884 (18.9%)	335/780 (42.9%)	**<0.001**
**Comorbidity**				
Hypertension	467/1672 (27.9%)	240/891 (26.9%)	227/781 (29.1%)	0.333
Diabetes	214/1672 (12.8%)	109/891 (12.2%)	105/781 (13.4%)	0.460
Other cardiovascular disease	159/1672 (9.5%)	86/891 (9.7%)	73/781 (9.3%)	0.832
Respiratory disease	84/1672 (5.0%)	51/891 (5.7%)	33/781 (4.2%)	0.162
Other disease	243/1672 (14.5%)	118/891 (13.2%)	125/781 (16.0%)	0.110
**Imaging features**				
**Position**				
Bilateral pulmonary	1375/1512 (90.9%)	699/791 (88.4%)	676/721 (93.8%)	**<0.001**
**Density**				
mGGO	1269/1487 (85.3%)	643/749 (85.8%)	626/738 (84.8%)	0.577
**Therapy**				
Antibiotics	441/1670 (26.4%)	149/887 (16.8%)	292/783 (37.3%)	**<0.001**
Antiviral	776/1674 (46.4%)	288/890 (32.4%)	488/784 (62.2%)	**<0.001**
Corticosteroids	105/1652 (6.4%)	16/884 (1.8%)	89/768 (11.6%)	**<0.001**
Traditional Chinese medicine treatment	1522/1669 (91.2%)	771/888 (86.8%)	751/781 (96.2%)	**<0.001**
Oxygen therapy	998/1641 (60.8%)	475/881 (53.9%)	523/760 (68.8%)	**<0.001**
Other therapy	109/1622 (6.7%)	32/871 (3.7%)	77/751 (10.3%)	**<0.001**
**Laboratory findings**				
Leucocyte count, ×10^9^ per L	5.60 (4.70, 6.80)	5.60 (4.80, 6.80)	5.50 (4.50, 6.90)	0.103
Lymphocyte count, ×10^9^ per L	1.56 (1.19, 1.92)	1.65 (1.30, 2.02)	1.43 (1.07, 1.81)	**<0.001**
Hemoglobin, g/L	125 (115, 136)	127 (116, 136)	123 (114, 134)	**<0.001**
Platelet count, × 10^9^ per L	226 (186, 279)	215 (181, 261)	241 (193, 305)	**<0.001**
NLR	2.18 (1.62, 3.01)	2.02 (1.58, 2.73)	2.39 (1.69, 3.42)	**<0.001**
CRP, mg/L	1.71 (0.68, 5.12)	1.22 (0.55, 3.03)	2.68 (0.91, 9.31)	**<0.001**
ALT, U/L	23.40 (14.60, 38.70)	21.20 (14.10, 33.78)	25.95 (15.80, 42.70)	**<0.001**
Albumin, g/L	38.30 (35.60, 40.63)	39.40 (36.90, 41.50)	36.90 (34.10, 39.50)	**<0.001**
BUN, mmol/L	4.32 (3.59, 5.22)	4.34 (3.61, 5.33)	4.27 (3.59, 5.15)	0.203
Creatinine, umol/L	64.00 (55.00,74.90)	63.90 (54.90, 75.00)	64.10 (55.00, 74.50)	0.941
Creatine kinase, U/L	50.10 (36.00, 71.25)	53.75 (38.85, 72.50)	45.20 (32.40, 67.40)	**<0.001**
LDH, U/L	170.30 (148.50, 201.20)	161.60 (142.50, 186.70)	185.20 (159.45, 228.95)	**<0.001**
Prothrombin time, s	12.72 (12.18, 13.40)	12.74 (12.23, 13.40)	12.71 (12.13, 13.40)	0.756
D-dimer, ug/L	0.35 (0.18, 0.64)	0.30 (0.17, 0.56)	0.41 (0.23, 0.82)	**<0.001**

Values are median (interquartile range) or number (percentage). *P*-values were calculated by Mann–Whitney U test or χ^2^ test, and bold represents significant differences between subgroups. COVID-19, coronavirus disease 2019; mGGO, mixed ground glass opacity; WBC, white blood cell; NLR, neutrophil-to-lymphocyte ratio; CRP, C reaction protein; ALT, alanine aminotransferase; BUN, blood urea nitrogen; LDH, lactate dehydrogenase

The variables with significant association assessed by the univariate logistic regression were shown in Additional file 1: Table S2. In the final multivariate logistic regression model, the number of clinical symptoms > 3 (Odds ratio [OR]: 1.86, 95% confidence interval [CI]: 1.47–2.36), maximum body temperature during hospitalization ≥ 37.3 ℃ (OR: 2.59, 95% CI: 1.99–3.36), abnormal platelet count (compared with below the lower limit of the normal, normal range, OR: 1.91, 95% CI: 1.03–3.55; increase, OR: 3.33, 95% CI: 1.58–7.02), increased levels of CRP (OR: 1.79, 95% CI: 1.37–2.34), increased LDH levels (OR: 1.62, 95% CI: 1.28–2.04) and decreased levels of albumin (OR: 2.01, 95% CI: 1.57–2.59) were independently associated with the length of hospital stay in moderate patients (Fig. 2a). The Hosmer and Lemeshow test of the final model showed an effective goodness-of-fit (*p* = 0.997). After excluding dead patients, the results were not materially altered (Additional file 1: Table S3).

**Fig. 3.**
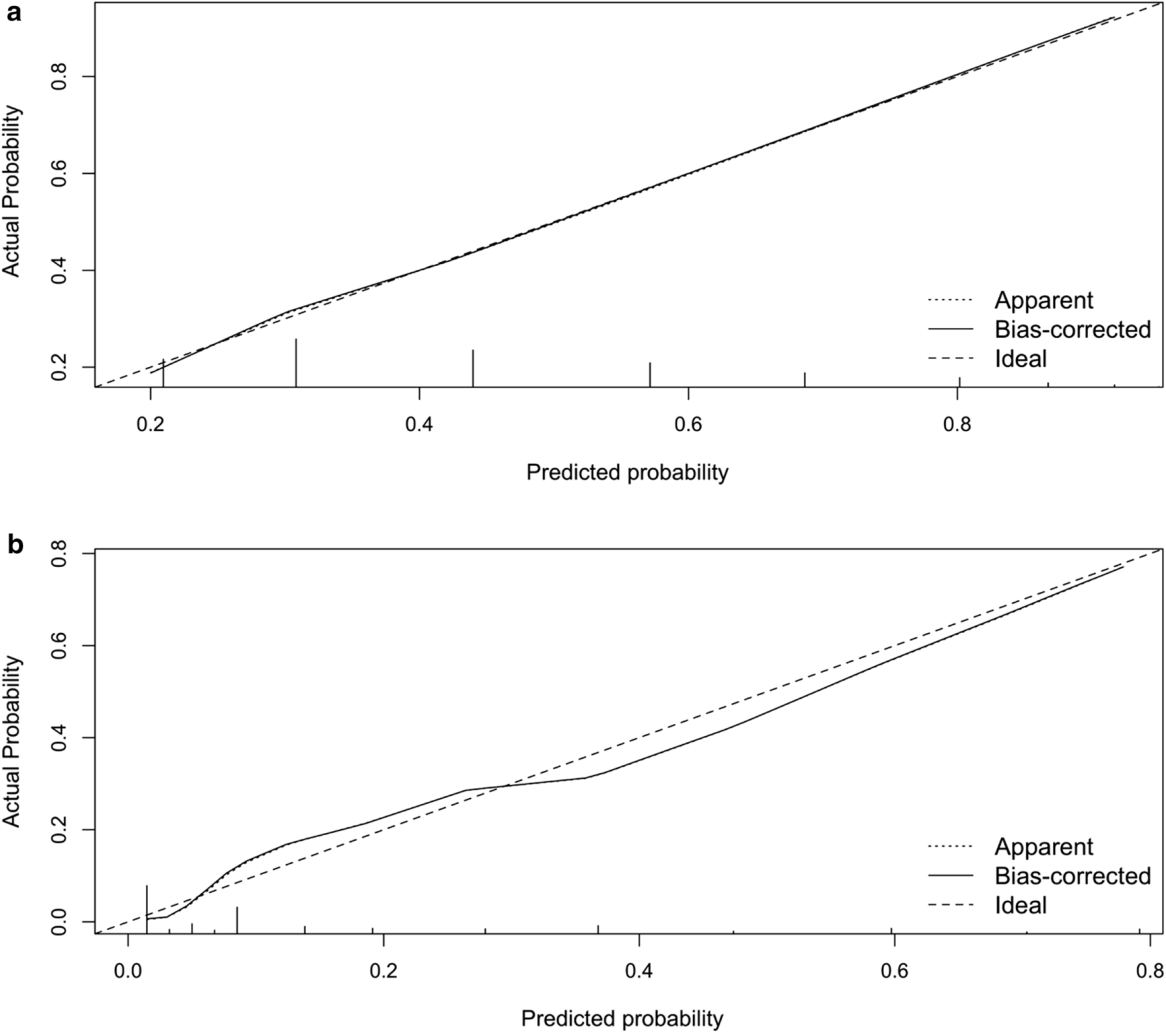
Calibration plots for predicting the probability of outcomes in COVID-19 patients. **a** STPCAL score for predicting hospitalization duration in moderate COVID-19 patients. **b** TRPNCLP score for predicting disease progression in severely ill COVID-19 patients. X-axis is predicted probability by risk scores, and y-axis is the actual probability of outcome events in our population. Dashed line represents the performance of the ideal scores. Dotted line is the apparent accuracy of our risk scores without overfitting correction. Solid line is the bootstrap-correction performance of our risk scores, representing dispersion estimation of future precision

In order to facilitate clinical application, we further built a risk scoring system to stratify the moderate patients with different length of stay. The risk scoring system was designated as STPCAL score including six variables: number of clinical symptoms, temperature, platelet count, CRP, albumin and LDH. The range of STPCAL score were 0 to 9 points. According to the STPCAL score, patients were classified into one of three risk categories for a longer hospital duration: low (Score 0‐1, median = 8 days, with less than 20.0% probabilities), intermediate (Score 2–6, median = 13 days, with 30.0–78.9% probabilities), high (Score 7–9, median = 19 days, with more than 86.5% probabilities) (Table 2). The bootstrapping AUC of the STPCAL score was 0.72 (95% CI: 0.69–0.75). The calibration plot demonstrated the adequate agreement between observed outcome events and predictions by our score with calibration-in-the-large of 0.001 and calibration slope of 0.998 (Fig. 3a).

**Table 2.** Risk categories by risk stratification scores among COVID-19 patients

	Categorization of risk			*p*-value
	Low	Intermediate	High	
**STPCAL score for hospitalization duration in moderate patients**				
Score	0 to 1	2 to 6	7 to 9	
Patient number (%)	239 (16.9)	1122 (79.1)	57 (4.0)	
Length of hospital stays, median (IQR)	8 (6, 12)	13 (8, 18)	19 (16, 25)	**<0.001**
Long-stay probability (%)^*^	< 20.0	30.0-78.9	> 86.5	
**TRPNCLP score for disease progression in severely ill patients**				
Score	0 to 5	6 to 11	12 to 16	
Patient number (%)	443 (74.7)	125 (21.1)	25 (4.2)	
Progression events, No. (%)	23 (5.2)	43 (34.4)	22 (88.0)	**<0.001**
Progression probability (%)^*^	< 12.7	18.6-69.1	> 77.9	

*P*-values were calculated by Kruskal-Wallis H test or χ^2^ test, and bold represents significant differences among the three subgroups. ^*^, probablities correspongding to each point were calculated by logistic regression model equation. COVID-19, coronavirus disease 2019; STPCAL, symptoms, temperature, platelet count, C-reactive protein, albumin, lactate dehydrogenase; TRPNCLP, temperature, respiratory disease, platelet count, neutrophil-to-lymphocyte ratio, creatinine, lactate dehydrogenase, prothrombin time

### Risk scoring system to predict disease progression of severely ill patients

Up to the end of the follow-up, there were still 17 severely ill patients who have not been discharged. According to the outcomes from the last follow-up, the 17 patients were classified in progression (n=2) and non-progression group (n=15). Baseline epidemiological and clinical characteristics of severely ill patients were shown in Table 3. The median age of patients in the non-progression group was 64 (56, 71.25) years, and 319 (51.3%) were male. The median age of patients was 68.5 (62, 75) years and 63.1% were male in the progression group. The patients were significantly older (*p* = 0.003), with more comorbidities such as history of other cardiovascular disease (*p* = 0.026), history of respiratory disease (*p* < 0.001), history of other disease (*p* = 0.005) in the progression group. Traditional Chinese medicine treatment (88.3%) was the most common, followed by oxygen therapy (83.2%) and antiviral therapy (58.7%). There were significantly higher levels of leucocyte count, NLR, CRP, BUN, creatinine, LDH, prothrombin time and D-dimer, but lower levels of lymphocyte count and albumin (*p* < 0.05) in patients with disease progression than those with non-progression.

**Table 3.** Demographic and clinical characteristics of severely ill patients with COVID-19

	Total (n = 744)	Non-progression (n = 622)	Progression (n = 122)	*p*-value
**Age (years)**	65 (56, 72)	64 (56, 71.25)	68.5 (62, 75)	**0.003**
≥ 60	505 (67.9%)	405 (65.1%)	100 (82.0%)	**<0.001**
**Gender**				
Male	396 (53.2%)	319 (51.3%)	77 (63.1%)	**0.017**
Female	348 (46.8%)	303 (48.7%)	45 (36.9%)	
**History of smoking**				
Yes	53/742 (7.1%)	42 (6.8%)	11/120 (9.2%)	0.347
**Clinical symptoms**				
Cough	544/743 (73.2%)	453 (72.8%)	91/121 (75.2%)	0.589
Fever	558/743 (75.1%)	469 (75.4%)	89/121 (73.6%)	0.667
Asthma or dyspnea	396/743 (53.3%)	325 (52.3%)	71/121 (58.7%)	0.195
Fatigue	436/743 (58.7%)	361 (58.0%)	75/121 (62.0%)	0.420
Myalgia	226/743 (30.4%)	185 (29.7%)	41/121 (33.9%)	0.365
Other features	205/743 (27.6%)	175 (28.1%)	30/121 (24.8%)	0.452
**Number of symptoms**	3 (2, 4)	3 (2, 5)	4 (2, 4)	0.068
> 3	335/743 (45.1%)	274 (44.1%)	61/121 (50.4%)	0.198
**Maximum body temperature ≥ 37.3 ℃**	314/719 (43.7%)	227/613 (37.0%)	87/106 (82.1%)	**<0.001**
**Comorbidity**				
Hypertension	328/742 (44.2%)	268 (43.1%)	60/120 (50.0%)	0.163
Diabetes	150/741 (20.2%)	126/621 (20.3%)	24/120 (19.2%)	0.942
Other cardiovascular disease	143/741 (19.3%)	111/621 (17.9%)	32/120 (26.7%)	**0.026**
Respiratory disease	89/743 (12.0%)	50 (8.0%)	39/121 (32.2%)	**<0.001**
Other disease	151/742 (20.4%)	115/621 (18.5%)	36/121 (29.8%)	**0.005**
**Imaging features**				
**position**				
Bilateral pulmonary	652/671 (97.2%)	573/591 (97.0%)	79/80 (98.8%)	0.364
**Density**				
mGGO	518/653 (79.3%)	463/578 (80.1%)	55/75 (73.3%)	0.173
**Therapy**				
Antibiotics	327/737 (44.4%)	233/619 (37.6%)	94/118 (79.7%)	**<0.001**
Antiviral	432/736 (58.7%)	349/618 (56.5%)	83/118 (70.3%)	**0.005**
Corticosteroids	186/737 (25.2%)	117/617 (19.0%)	69/120 (57.5%)	**<0.001**
Traditional Chinese medicine treatment	639/724 (88.3%)	548/613 (89.4%)	91/111 (82.0%)	**0.026**
Oxygen therapy	613/737 (83.2%)	499/618 (80.7%)	114/119 (95.8%)	**<0.001**
Other therapy	203/733 (27.7%)	128/615 (20.8%)	75/118 (63.6%)	**<0.001**
**Laboratory findings**				
Leucocyte count, × 10^9^ per L	6.00 (4.80, 7.80)	5.80 (4.73, 7.20)	8.30 (5.70, 11.10)	**<0.001**
Lymphocyte count, × 10^9^ per L	1.32 (0.89, 1.71)	1.42 (0.98, 1.78)	0.84 (0.51, 1.20)	**<0.001**
Hemoglobin, g/L	120 (109, 132)	121 (110, 132)	119 (102, 130)	0.271
Platelet count, × 10^9^ per L	224 (168, 274)	225 (177, 271)	216 (132, 292)	0.156
NLR	2.85 (1.88, 5.25)	2.59 (1.80, 3.94)	7.74 (3.74, 17.11)	**<0.001**
CRP, mg/L	4.31 (1.39, 22.46)	3.26 (1.15, 12.26)	51.99 (11.70, 104.77)	**<0.001**
ALT, U/L	21.80 (14.80, 36.83)	21.45 (14.63, 35.90)	26.70 (15.38, 46.98)	0.119
Albumin, g/L	36.40 (32.60, 39.30)	37.00 (33.60, 39.60)	32.70 (29.18, 36.03)	**<0.001**
BUN, mmol/L	4.59 (3.63, 6.12)	4.44 (3.57, 5.68)	6.34 (4.29, 9.08)	**<0.001**
Creatinine, umol/L	64.30 (54.30, 76.75)	63.20 (53.50, 75.20)	68.75 (56.70,89.58)	**0.002**
Creatine kinase, U/L	45.91 (31.68, 70.43)	45.60 (31.83, 66.65)	48.20 (30.85, 98.60)	0.596
LDH, U/L	200.10 (166.00, 258.60)	192.10 (162.95, 237.05)	296.25 (208.78, 430.08)	**<0.001**
Prothrombin time, s	12.97 (12.30, 13.90)	12.86 (12.24, 13.68)	14.10 (12.69, 15.45)	**<0.001**
D-dimer, ug/L	0.68 (0.33, 1.54)	0.58 (0.31, 1.25)	1.95 (0.81, 4.73)	**<0.001**

Values are median (interquartile range) or number (percentage). P-values were calculated by Mann-Whitney U test or χ^2^ test, bold represents significant differences between subgroups. COVID-19, coronavirus disease 2019; mGGO, Mixed Ground Glass Opacity; NLR, neutrophil-to-lymphocyte ratio; CRP, C-reactive protein; ALT, alanine aminotransferase; BUN, blood urea nitrogen; LDH, lactate dehydrogenase.

The variables with significant association assessed by the univariate logistic regression were shown in Additional file 1: Table S4. In the final multivariate logistic regression model, we found that the maximum body temperature during hospitalization ≥ 37.3 ℃ (OR: 5.24, 95% CI: 2.70–10.16), history of respiratory diseases (OR: 2.43, 95% CI: 1.20–4.92), decreased platelet count (OR: 2.39, 95% CI: 1.04–5.47), NLR > 5.00 (OR: 3.31, 95% CI: 1.74–6.30), increased levels of creatinine (OR: 2.94, 95% CI: 1.44–6.02), LDH (OR: 1.59, 95% CI: 1.16–2.18) and prothrombin time (OR: 2.51, 95% CI: 1.19–5.27) were independently associated with disease progression in severely ill patients (Fig. 2b). The goodness of fit of the final model was acceptable according to Hosmer and Lemeshow test (*p* = 0.898).

We used beta coefficients of the above significant factors to construct a relative weighted score system, named as TRPNCLP score (temperature, respiratory disease, platelet count, NLR, creatinine, LDH, and prothrombin time score). The AUC of TRPNCLP score by bootstrapping was 0.88 (95% CI: 0.85–0.91), which was higher than that of MuLBSTA score (0.76, 95% CI: 0.73–0.79, *p* < 0.001). Similar differences were observed by NRI and IDI, indicating that TRPNCLP score had a significantly better reclassification than MuLBSTA score (Table 4). Furthermore, the TRPNCLP score was well-calibrated with calibration-in-the-large and calibration slope equal to 0.004 and 1.002, respectively (Fig. 3B). The range of TRPNCLP score were 0 to 16 points. We further classified the TRPNCLP score into 3 levels to stratify the risk of disease progression: low risk (Score 0–5, n = 23 or 5.2%, with less than 12.7% probabilities), intermediate risk (Score 6–11, n = 43 or 34.4%, with 18.6–69.1% probabilities), and high risk (Score 12–16, n = 22 or 88.0%, with more than 77.9% probabilities) (Table 2).

**Table 4.** The comparison of TRPNCLP with MuLBSTA score among severely ill COVID-19 patients

Models	AUC (95% CI)	*P* value	Cut-off value	Sensitivity (95% CI)	Specificity (95% CI)	PPV (95% CI)	NPV (95% CI)	NRI (%) (95% CI)	*P* value	IDI (%) (95%CI)	*P* value
MuLBSTA	0.76 (0.73–0.79)	Ref	12	0.56 (0.43–0.68)	0.81 (0.77–0.84)	0.29 (0.23–0.35)	0.93 (0.91–0.95)	Ref	Ref	Ref	Ref
TRPNCLP	0.88 (0.85–0.91)	**< 0.001**	5	0.71 (0.59–0.82)	0.83 (0.80–0.87)	0.37 (0.32–0.43)	0.95 (0.93–0.97)	17.42 (4.31–30.53)	**0.009**	17.42 (4.21–30.62)	**0.009**

Bold indicates *p* < 0.05.

AUC, area under receiver operator characteristic (ROC) curve; 95% CI, 95% confidence interval; PPV, positive predictive value; NPV, negetive predictive value; NRI, net reclassification improvement; IDI, integrated discrimination improvement; MuLBSTA, multilobular infiltration, hypo-lymphocytosis, bacterial coinfection, smoking history, hypertension and age; TRPNCLP, temperature, respiratory disease, platelet count, neutrophil-to-lymphocyte ratio, creatinine, lactate dehydrogenase, prothrombin time.

## Discussion

In this cohort study, we identified risk factors for hospitalization duration and disease progression in patients from a large special hospital of COVID-19 in Wuhan, China. In particular, more clinical symptoms, abnormal platelet count, higher CRP, lower albumin, higher LDH on admission and higher body temperature during hospitalization were significantly associated with long-stay duration in moderate patients. History of respiratory disease, lower platelet count, higher NLR, higher creatinine, higher LDH, prolonged PT, and higher body temperature were associated with increased risk of disease progression in severely ill patient. Additionally, we built two easy-to-use risk stratification score systems that can be used by clinicians, named as STPCAL and TRPNCLP, to estimate the risk of hospital stay duration and disease progression, respectively.

CRP is an important biomarker to reflect cell injury, inflammation and tissue damage. The increase of CRP may indicate the state of inflammatory reaction and the degree of damage to the immune system caused by viral infection. Several studies have found that CRP levels were associated with COVID-19 severity [13, 14]. Furthermore, a preprint study has also shown that CRP is one of the earliest biomarkers that changes to reflect physiological complications and could be used as an effective biomarker for predicting progression of COVID-19 infection [15]. The deficiency of nutritional intake, consumption of albumin by the synthesis of acute inflammatory protein, and the abnormal distribution of albumin caused by pulmonary exudation are reflecting by the decrease of albumin [16]. A recent meta-analysis has shown decreased serum albumin level has been associated with severe COVID-19 and mortality. Low albumin level can help to early recognition of severe COVID-19 [17]. Our findings provided an important piece of evidence that both elevated CRP and decreased albumin were independently associated with hospital long-stay in patients with moderate COVID-19.

LDH represents the glucose metabolism of body tissue, high LDH levels are associated with cell damage occurring in various diseases, including inflammatory pulmonary disorders. Up to date, more and more convincing evidence links LDH as a biomarker to the development and severity of COVID-19 infection [18]. Han et al. reported that LDH was an important indicator to reflect the disease severity of COVID-19 patients [19]. They found that LDH was positively correlated to the indicators of inflammation, heart and liver function damage, but negatively correlated with lymphocyte count. Several studies suggest that LDH was a predictor of COVID-19 progression and mortality [13, 20]. Our study further demonstrated the role of LDH in COVID-19, suggesting that LDH could be an auxiliary marker predicting a longer hospital stay and disease progression.

Platelet count is a simple and easy-to-use biomarker in clinical practice. Current studies have shown that a variety of cytokines, including IL-3, IL-6, IL-9, and IL-11, can promote the production of megakaryocytes and release of platelet. However, severe infections could cause secondary thrombocytopenia, such as disseminated intravascular coagulation (DIC), which is associated with significant bleeding manifestations and more common in fatal outcomes. Interestingly, we found that among moderate patients, normal range and increased platelets levels in patients predicted longer average hospital stay compared to patients with thrombocytopenia. Qu et al. reported that patients with platelet peaks have a longer average hospital stay, which is consistent with our finding [21]. Increased platelets activated by excessive inflammation affect abnormal coagulation state and faster aggregation, leading to thrombotic disease [22]. Moreover, pulmonary micro-thrombosis disturbs the blood oxygen transport to reduce lung function of the patients, which may be related to the longer course of the disease. Different with what was found in moderate patients, we found that lower platelet count could predict the progression in severely ill patients. Lower platelet count was associated with disease severity score and considered to be a risk factor for death in patients with severe acute respiratory syndrome (SARS) [23]. Studies also reported that thrombocytopenia could increase the risk of severe, in-hospital mortality or bleeding complications during hospitalization of COVID-19 and, thus, should serve as an indicator of deterioration during hospitalization [24–26]. Recently, data suggested that coagulation disorder caused by COVID-19 may be different from common infection-induced DIC. Increasing in circulating biomarkers may directly bind to platelet receptors, followed by platelet hyperactivation and aggregation, during such hyperactivation, platelet count is lower. Hyperresponsive platelets could contribute to the cytokine storm, while platelets were excessively consumed in severe COVID-19 patients due to the activation of coagulation pathway by cytokine storm, resulting in microcirculatory coagulation disorders and forming a vicious circle [27, 28]. Therefore, we suspected that inflammation levels caused by infection leads to slightly activation of platelets during early-stage COVID-19, thrombocytopenia representing derangement of platelet function may be associated with hematopoietic inhibition, pulmonary damage, secondary infections and increased consumption of megakaryocytes and platelet during later-stage of the progression of the disease, reflecting conditions that are more prone to progression [21, 27]. But for moderate patients, the clinical value of lower platelet count predicting shorter hospital stay needs to be explored by further studies.

Furthermore, prothrombin time could be used for early diagnoses of DIC. Compared with survivors, non-survivors had longer prothrombin time [29, 30]. Increasing prothrombin time has been found to be significantly correlation with disease progression of COVID-19 [31]. Prolonged prothrombin time may indicate excessive consumption of coagulating factors. In our study, prolonged prothrombin time has been identified as a risk factor to predict disease progression. Our findings confirmed that blood coagulation dysfunction may play a central role in the deterioration of the disease, and suggested that patients with the above coagulation-related indexes should be closely monitored [32].

Several studies revealed the differences of baseline leucocyte count among patients with different clinical types of COVID-19. Compared with survivors, non-survivors had more significantly increased leucocyte count [14, 30], which may be driven by elevated neutrophils. Li et al. found that higher neutrophil and lower lymphocyte count could predict in-hospital mortality for COVID-19 patients [25]. NLR is an effective index reflecting the imbalance between neutrophil count and lymphocyte count, which is related to multiple organ injury. Elevated NLR may indicate the immunologic abnormality, and was related with severity of COVID-19 and in-hospital death [33]. Furthermore, Yang et al. identified NLR as discriminator to improve prediction for poor clinical outcome in COVID-19 patients [34]. Our findings were consistent with these lines of evidence, which suggested that NLR could be an important early prediction marker for disease progression in severely ill patients.

SARS-CoV-2 may also invade renal tubular epithelial cells though angiotensin converting enzyme II (ACE2) receptor, which expressed not only in respiratory organs but also in the kidney [35]. Therefore, researchers began to be concerned about the renal function of COVID-19 patients. Creatinine, as a commonly used clinical index, reflects the state of renal function. One meta-analysis showed the significant association of elevated creatinine with severe or fatal patients [14]. Cheng et al. reported that higher serum creatinine was a risk factor of in-hospital mortality of COVID-19 patients [36]. Patients with elevated plasma creatinine are more likely to be admitted to ICU and develop acute renal injury, which was strongly related to increased mortality [36, 37]. Our finding, consistent with the previous results, demonstrated that higher creatinine was involved in the disease progression.

Cardiovascular diseases may be a significant determinant of disease progression among COVID-19 patients. Some studies suggest that screening for acute coronary syndrome may be underestimated in the context of COVID-19 outbreak. Besides, unstable hemodynamic and pro-inflammatory state caused by acute respiratory failure of COVID-19 may promote the occurrence of acute coronary syndrome and lead to a poor prognosis of patients [38]. Li et al. reported that there was a significant positive association between cardiovascular disease and in-hospital mortality of COVID-19. However, this result was obtained from an unadjusted meta-analysis [39]. In our study, we found that severely ill patients with underlying cardiovascular comorbidities were more likely to suffer a poor prognosis. However, Cardiovascular disease did not as an independent risk factor for prognosis in multivariate analysis. Its role may have been offset by some other biomarkers. Besides, studies have shown the role of cardiac troponin in worsening clinical outcomes of patients [40, 41]. However, our research failed to collect the results of cardiac troponin test, and more reliable data are needed to warrant the relationship between it and the prognosis of COVID-19. Several studies also suggested that features derived from CT scoring were predictors for prognosis of COVID-19 [4]. However, radiological features were not included in our final prediction models. Considering the reason, it may be that patients with moderate, severe and critical type were separated into two groups, and the radiological characteristics of patients with the same clinical type may not be significantly different in our study. Besides, this study was based on a single center with limited population representation.

Currently, prediction models for COVID-19 related mortality have been published [20, 42]. Several studies have focused on identifying risk factors related to the progression to severe or critical disease in patients with COVID-19, using a nomogram to predict the risk of disease progression visually [13, 43, 44]. However, except for the small sample size, Wynants et al. pointed out that most of these models exclude patients who are still in hospital by the end of the study and had high risk of over-fitting [4]. Furthermore, most of published studies have not concerned the differences in clinical endpoints between moderate and severely ill patients. Thus, considering the balance between practicality and accuracy, we constructed simplified prediction scores for risk stratification of the hospital stay and disease progression for patients with different clinical types, respectively. Scholars have constructed a score named MuLBSTA for the poor prognosis of viral pneumonia, which was in line with COVID-19 patients [30]. But as shown by the results of comparison of AUC, sensitivity, specificity, NRI and IDI, MuLBSTA scores are not as good as our TRPNCLP score. The prediction scores we constructed performed well with good discrimination and calibration. In addition, according to the score, patients can be divides into low-, medium- and high-risk groups to guide the clinical decision. For example, a 67-year old severely ill patient with maximum body temperature of 37.4 ℃, respiratory disease, platelet count level of 164 x10^9^/L, NLR of 20.6, creatinine of 56.40 umol/L, LDH of 361.90 U/L, and PT of 15.81 s. According to TRPNCLP score, this case receives a total of 12 points (4 points for temperature, 2 for respiratory disease, 0 for platelet count, 3 for NLR levels, 0 for creatinine, 1 for LDH, and 2 for PT), and would be predicted to have a high-risk of progression. The risk stratification scores constructed in this study might help clinicians classify patients accurately in the face of limited health resources and improve the survival rate of severe and critical patients.

To the best of our knowledge, this study is the largest cohort study on subgroup patients with moderate and severe COVID-19. However, the current study has several limitations. Firstly, it is a single-center study. Although the discrimination and calibration of prediction models were internally verified by a bootstrap method, the models are needed to be verified in independent external populations. Secondly, the roles of some biomarkers (such as IL-6, cTn and procalcitonin) may be ignored or underestimated in the predicting models because data were extracted from a real-world clinical patient cohort, and not all laboratory tests were done in all patients.

## Conclusions

In summary, we found that moderate COVID-19 patients with more clinical symptoms, elevated platelet count, CRP and LDH, lower albumin at admission and higher body temperature during hospitalization had a high probability of longer hospital stay; severely ill patients having a history of respiratory disease, higher NLR, creatinine, LDH, and PT, lower platelet count at admission, and higher body temperature during hospitalization had a higher risk for disease progression. Using these clinical features and routine blood test indexes, we constructed two easy-to-use risk stratification score systems, named as STPCAL and TRPNCLP, to predict hospitalization duration and disease progression, respectively. In the current COVID-19 pandemic and the absence of specific remedies, early risk prediction and stratification will contribute to precise management of patients and effective use of limited health resources.

### Reporting guideline statement

We present this article in accordance with the STROBE reporting checklist.

## Declarations

## Supplementary Information

**Additional file 1**. Statistical analysis for indicators of COVID-19 hospitalization duration, progression and associated factors.

## Data Availability

The data that support the findings of this study are available from the corresponding author on reasonable request.

## Abbreviations

ALT: alanine aminotransferase
AUC: area under the ROC curve
BUN: Blood urea nitrogen
CI: Confidence interval
COVID-19: Coronavirus disease 2019
CRP: C-reactive protein
DIC: Disseminated intravascular coagulation
ICU: Intensive Care Unit
LDH: lactate dehydrogenase
NLR: neutrophil-to-lymphocyte ratio
OR: Odds ratio
ROC: receiver operator characteristic
SARS-CoV-2: severe acute respiratory syndrome coronavirus 2
VIF: variance inflation factor
