# Supplementary material for: Risk stratification scores for hospitalization duration and disease progression in moderate and severe patients with COVID-19
Source: BMC Pulm Med. 2021 Apr 14;21:120. doi: 10.1186/s12890-021-01487-6 (PMC8045569; doi:10.1186/s12890-021-01487-6)
Supplement: Supplementary file 1 — Additional file 1. Statistical analysis for indicators of COVID-19 hospitalization duration, progression and associated factors. [file 12890_2021_1487_MOESM1_ESM.docx]

**Risk stratification scores for hospitalization duration and disease progression in moderate and severe patients with COVID-19**

Jiaqi Huang^1, #^, Yu Xu^2, #^, Bin Wang^2, #^, Ying Xiang^1^, Na Wu^1^, Wenjing Zhang^2^, Tingting Xia^1^, Zhiquan Yuan^1^, Chengying Li^1^, Xiaoyue Jia^1^, Yifan Shan^1^, Menglei Chen^1^, Qi Li^2 *^, Li Bai^2 *^, Yafei Li^1, *^

^1^ *Department of Epidemiology, College of Preventive Medicine, Army Medical University (Third Military Medical University), Chongqing 400038, People’s Republic of China*

^2^ *Department of Respiratory and Critical Care Medicine, the Second Affiliated Hospital of The Army Medical University, Chongqing 400037, People’s Republic of China*

^#^ These authors contributed equally to this work.

^*^ These authors jointly directed the project.

**Supplementary materials**

**Table S1.** **Normal ranges for clinical laboratory testing indicators.**

**Table S2.** **Factors associated with longer hospital stay in moderate patients with COVID-19 by univariate logistic regression analysis.**

**Table S3. Factors associated with longer hospital stay in moderate patients with COVID-19 by sensitivity analysis.**

**Table S4.** **Factors associated with disease progression in severely ill patients with COVID-19 by univariate logistic regression analysis.**

| **Table S1 Normal ranges for clinical laboratory testing indicators** | |
| --- | --- |
| Laboratory chemistries | Normal ranges |
| Leucocyte count (x10ˆ9 per L) | 3.5-9.5 |
| Lymphocyte count (x10ˆ9 per L) | 1.1-3.2 |
| Hemoglobin (g/L) |  |
| Male | 130-175 |
| Female | 115-150 |
| Platelet count (x10ˆ9 per L) | 125-350 |
| CRP (mg/L) | 0-4 |
| ALT (U/L) |  |
| Male | 9-50 |
| Female | 7-40 |
| Albumin (g/L) | 40-55 |
| BUN (mmol/L) |  |
| Male (age < 60 years) | 3.1-8.0 |
| Male (age 60-80 years) | 3.6-9.5 |
| Female (age < 60 years) | 2.6-7.5 |
| Female (age 60-80 years) | 3.1-8.8 |
| Male / Female (age > 80 years) | 2.6-7.5 |
| Creatinine (umol/L) |  |
| Male (age < 60 years) | 57-97 |
| Male (age 60-80 years) | 57-111 |
| Female (age < 60 years) | 41-73 |
| Female (age 60-80 years) | 41-81 |
| Male / Female (age > 80 years) | 41-73 |
| Creatine kinase (U/L) |  |
| Male | 24-190 |
| Female | 24-170 |
| LDH (U/L) | 120-250 |
| PT (s) | 9.2-15 |
| D-dimer (mg/L) | 0-0.55 |

CRP, C-reactive protein; ALT, Aspartate aminotransferase; BUN, blood urea nitrogen; LDH, Lactate dehydrogenase; PT, prothrombin time.

| **Table S2 Factors associated with longer hospital stay in moderate patients with COVID-19 by univariate logistic regression analysis** | | | |
| --- | --- | --- | --- |
|  | Univariate logistic regression | | |
|  | Beta-coefficients | OR (95%CI) | *p*-value |
| Age ( ≥ 60 years vs < 60 years) | 0.45 | 1.57 (1.30-1.91) | **<0.001** |
| Number of clinical symptoms ( > 3 vs ≤ 3) | 0.71 | 2.03 (1.67-2.48) | **<0.001** |
| Maximum body temperature ( ≥ 37.3 ℃ vs < 37.3 ℃) | 1.17 | 3.23 (2.59-4.02) | **<0.001** |
| Position (bilateral vs unilateral pulmonary) | 0.68 | 1.98 (1.36-2.87) | **<0.001** |
| Lymphocyte count, per one-unit increase | -0.67 | 0.51 (0.43-0.62) | **<0.001** |
| Hemoglobin, per one-unit increase | -0.01 | 0.99 (0.99-1.00) | **0.003** |
| Platelet count, per one-unit increase | 0.01 | 1.01 (1.00-1.01) | **<0.001** |
| NLR, per one-unit increase | 0.14 | 1.15 (1.09-1.22) | **<0.001** |
| CRP, per one-unit increase | 0.02 | 1.02 (1.02-1.03) | **<0.001** |
| ALT, per one-unit increase | 0.01 | 1.01 (1.00-1.01) | **<0.001** |
| Albumin, per one-unit increase | -0.17 | 0.85 (0.82-0.87) | **<0.001** |
| LDH, per one-unit increase | 0.01 | 1.01 (1.00-1.01) | **<0.001** |
| D-dimer, per one-unit increase | 0.20 | 1.22 (1.08-1.37) | **0.001** |

OR (95% CI) and p-values were calculated using an univariate logistic regression analysis. Bold indicates p < 0.05. COVID-19, coronavirus disease 2019; OR, odds ratio; 95% CI, 95% confidence interval; NLR, neutrophil-to-lymphocyte ratio; CRP, C-reactive protein; ALT, alanine aminotransferase; LDH, lactate dehydrogenase.

| **Table S3 Factors associated with longer hospital stay in moderate patients with COVID-19 by sensitivity analysis** | | | | |
| --- | --- | --- | --- | --- |
|  |  | Multivariate logistic regression^*^ | | |
|  |  | Beta-coefficients | OR (95%CI) | *p*-value |
| Number of clinical features |  |  |  |  |
| ≤ 3 |  |  | Reference |  |
| > 3 |  | 0.62 | 1.85 (1.46-2.35) | **<0.001** |
| Temperature |  |  |  |  |
| < 37.3 ℃ |  |  | Reference |  |
| ≥ 37.3 ℃ |  | 0.95 | 2.59 (1.99-3.37) | **<0.001** |
| Platelets count |  |  |  |  |
| Normal range |  | 0.65 | 1.92 (1.04-3.57) | **0.038** |
| < Lower limits of normal |  |  | Reference |  |
| > Upper limits of normal |  | 1.21 | 3.35 (1.59-7.06) | **0.002** |
| CRP |  |  |  |  |
| ≤ Upper limits of normal |  |  | Reference |  |
| > Upper limits of normal |  | 0.58 | 1.79 (1.37-2.34) | **<0.001** |
| Albumin |  |  |  |  |
| ≥ Lower limits of normal |  |  | Reference |  |
| < Lower limits of normal |  | 0.70 | 2.01 (1.56-2.58) | **<0.001** |
| LDH |  |  |  |  |
| ≤ Upper limits of normal |  |  | Reference |  |
| > Upper limits of normal |  | 0.50 | 1.65 (1.30-2.09) | **<0.001** |

^*^OR (95% CI) and p-values were calculated using an multivariate logistic regression analysis by excluding death patients (n = 6). Bold indicates p < 0.05. COVID-19, coronavirus disease 2019; OR, odds ratio; 95% CI, 95% confidence interval; CRP, C reaction protein; LDH, lactate dehydrogenase.

| **Table S4 Factors associated with disease progression in severely ill patients with COVID-19 by univariate logistic regression analysis** | | | |
| --- | --- | --- | --- |
|  | Univariate logistic regression | | |
|  | Beta-coefficients | OR (95%CI) | *p*-value |
| Age ( ≥ 60 years vs < 60 years) | 0.89 | 2.44 (1.49-3.98) | **<0.001** |
| Sex (Female vs Male) | -0.49 | 0.62 (0.41-0.92) | **0.017** |
| Maximum body temperature ( ≥ 37.3 ℃ vs < 37.3 ℃) | 2.05 | 7.79 (4.62-13.13) | **<0.001** |
| Other cardiovascular diseases | 0.51 | 1.67 (1.06-2.63) | **0.027** |
| Respiratory disease | 1.69 | 5.44 (3.37-8.78) | **<0.001** |
| Other diseases | 0.62 | 1.86 (1.20-2.89) | **0.006** |
| Leucocyte count, per one-unit increase | 0.26 | 1.30 (1.21-1.39) | **<0.001** |
| Lymphocyte count, per one-unit increase | -1.45 | 0.24 (0.16-0.36) | **<0.001** |
| Platelet count, per one-unit increase | -0.01 | 1.00 (1.00-1.01) | **0.049** |
| NLR, per one-unit increase | 0.15 | 1.17 (1.12-1.21) | **<0.001** |
| CRP, per one-unit increase | 0.02 | 1.02 (1.02-1.03) | **<0.001** |
| ALT, per one-unit increase | 0.01 | 1.01 (1.00-1.01) | **0.003** |
| Albumin, per one-unit increase | -0.18 | 0.83 (0.79-0.87) | **<0.001** |
| BUN, per one-unit increase | 0.19 | 1.21 (1.13-1.29) | **<0.001** |
| Creatinine, per one-unit increase | 0.01 | 1.00 (1.00-1.01) | **0.013** |
| Creatine kinase, per one-unit increase | 0.01 | 1.00 (1.00-1.01) | **0.002** |
| LDH, per one-unit increase | 0.01 | 1.01 (1.00-1.01) | **<0.001** |
| PT, per one-unit increase | 0.44 | 1.55 (1.34-1.78) | **<0.001** |
| D-dimer, per one-unit increase | 0.25 | 1.29 (1.18-1.41) | **<0.001** |

OR (95% CI) and p-values were calculated using an univariate logistic regression analysis. Bold indicates p < 0.05. COVID-19, coronavirus disease 2019; OR, odds ratio; 95% CI, 95% confidence interval; NLR, neutrophil-to-lymphocyte ratio; CRP, C reaction protein; ALT, alanine aminotransferase; BUN, blood urea nitrogen; LDH, lactate dehydrogenase; PT, prothrombin time.
